# Consequences of the COVID-19 Outbreak Lockdown on Non-Viral Infectious Agents as Reported by a Laboratory-Based Surveillance System at the IHU Méditerranée Infection, Marseille, France

**DOI:** 10.3390/jcm10153210

**Published:** 2021-07-21

**Authors:** Lanceï Kaba, Audrey Giraud-Gatineau, Marie-Thérèse Jimeno, Jean-Marc Rolain, Philippe Colson, Didier Raoult, Hervé Chaudet

**Affiliations:** 1 IHU Méditerranée Infection, 19-21 Boulevard Jean Moulin, 13005 Marseille, France; lancekaba@yahoo.fr (L.K.); audrey.giraud.gatineau@gmail.com (A.G.-G.); jean-marc.rolain@univ-amu.fr (J.-M.R.); philippe.colson@univ-amu.fr (P.C.); didier.raoult@gmail.com (D.R.); 2 Aix Marseille Université, Institut de Recherche pour le Développement (IRD), Assistance Publique-Hôpitaux de Marseille (AP-HM), Service de Santé des Armées (SSA), VITROME, 13005 Marseille, France; 3 Institut Supérieur des Sciences et de Médecine Vétérinaire (ISSMV) de Dalaba, BP 09 Dalaba, Guinea; 4 French Armed Forces Center for Epidemiology and Public Health (CESPA), Service de Santé des Armées (SSA), 13014 Marseille, France; 5 Assistance Publique-Hôpitaux de Marseille (AP-HM), 13005 Marseille, France; marie-therese.jimeno@ap-hm.fr; 6 Aix-Marseille Université, Institut de Recherche pour le Développement (IRD), Assistance Publique-Hôpitaux de Marseille (AP-HM), MEPHI, 27 Boulevard Jean Moulin, 13005 Marseille, France

**Keywords:** syndromic surveillance, clinical microbiology laboratory, epidemiology, lockdown, COVID-19, diversity, wild

## Abstract

The objective of this paper is to describe the surveillance system MIDaS and to show how this system has been used for evaluating the consequences of the French COVID-19 lockdown on the bacterial mix of AP-HM and the antibiotic resistance. MIDas is a kind of surveillance activity hub, allowing the automatic construction of surveillance control boards. We investigated the diversity and resistance of bacterial agents from respiratory, blood, and urine samples during the lockdown period (from week 12 to 35 of 2020), using the same period of years from 2017 to 2019 as control. Taking into account the drop in patient recruitment, several species have exhibited significant changes in their relative abundance (either increasing or decreasing) with changes up to 9%. The changes were more important for respiratory and urine samples than for blood samples. The relative abundance in respiratory samples for the whole studied period was higher during the lockdown. A significant increase in the percentage of wild phenotypes during the lockdown was observed for several species. The use of the MIDaS syndromic collection and surveillance system made it possible to efficiently detect, analyze, and follow changes of the microbiological population as during the lockdown period.

## 1. Introduction

The Hospital University Institute Méditerranée Infection (IHU-MI) hosts the clinical microbiology and virology laboratory for all four of the public university hospitals of Marseille (AP-HM) that perform the diagnosis of infectious agents including bacteria, microscopic fungi, parasites, and viruses. Since 2013, it has implemented and improved five syndromic epidemiological surveillance sub-systems that use its laboratory results from a single collection and an analysis system named MIDaS (Mediterranée Infection Data Warehousing and Surveillance). Besides traditional surveillance based on patients’ clinical diagnoses of notifiable infectious diseases, syndromic surveillance uses data about analysis requests from clinicians’ prescriptions and laboratory’s tests results, as well as other laboratory markers, through innovative approaches.

In December 2019, Wuhan in the Hubei province became the epicenter of the spread of a new emerging pathogen called SARS-CoV-2. It spreads rapidly to other continents, a pandemic being declared officially in March 2020 (https://www.who.int/emergencies/diseases/novel-coronavirus-2019/interactive-timeline/, accessed on 27 May 2021). For controlling the rapid spread of this virus, the French government implemented various measures including the closure of schools, cultural centers, and socialization places such as bars and restaurants [1,2], before finally promulgating a total lockdown one week later, from 16 March 2020 [3] to 11 May 2020 (week 12 to 19). Many European, American, and Asian countries have made the same choice with a more or less strict lockdowns [4]. Several studies have already assessed the effectiveness and impact of the diverse consequences of lockdowns in various fields such as the medical, economic, or sociological fields [5,6,7,8]. They showed that lockdown measures led to major alterations of patient cares in hospital settings, especially those with chronic non-communicable diseases such as hypertension, diabetes, mental depression, etc. [9]. In the UK, a 71% decrease in blood counts was reported in the first four weeks of containment, and 57% fewer patients were sent for specialist hematology review [10]. However, to our knowledge, the microbiological impact of lockdown measures has not been yet studied.

The objective of this paper is to describe the current organization of the surveillance system and to show how it has been used for evaluating the consequences of the French lockdown on bacterial identifications in the AP-HM, as well as its influence on antibiotic resistance.

## 2. Materials and Methods

### 2.1. MIDaS, an Epidemiological Hub

**Figure 1:**
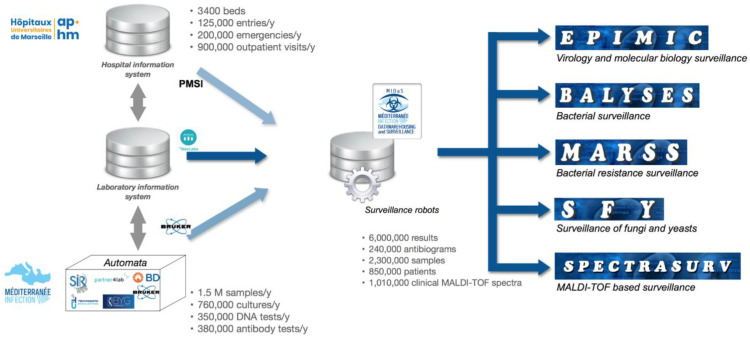
Structure of Méditerranée Infection Data warehousing and Surveillance (MIDaS).

The MIDaS system can be considered as a surveillance activity hub, hosting data coming from the hospital information system, the analysis automata, and several other sources such as the taxonomy database maintained by NCBI/GenBank (https://www.ncbi.nlm.nih.gov/Taxonomy/Browser/wwwtax.cgi, accessed on 27 May 2021) and the demographical data coming from the Institut national de la statistique et des études économiques (https://www.insee.fr/fr/statistiques, accessed on 27 May 2021). Before their analysis, data are preprocessed depending on their nature (bacterial identification, molecular biology, antiobiotic resistance, etc.) for specific aggregations or expert processing, as the identification of resistance phenotype by expert rules. Raw data and preprocessing results are then systematically analyzed and presented by statistical automata in search of statistical aberrations within time series [11] (e.g., a significant increase suggesting a possible health concern or a disease outbreak). All significant increases are automatically documented by the system, including the sampling profiling, the search for atypical antibiograms, and the mapping of cases. Analysis results are dynamically presented using tailored control boards on an internal dedicated website, and are discussed each week during a staff meeting that is attended by biologists, clinicians, and epidemiologists. During this staff meeting, in silico investigations (notably additional comparisons and cross-referencing of data) can be used. Furthermore, epidemiological investigations can be initiated if the investigation confirms the alarm and, if required, an epidemiological alert may be simultaneously transmitted to the health institutions concerned including the Regional Health Agency (Agence Régionale de Santé, ARS, Marseille, France) and the Infection Control Committee (Comité de Lutte contre les Infections nosocomiales, CLIN, Marseille, France). The surveillance results are also disseminated weekly through the IHU Méditerranée Infection website As a surveillance activity hub, this system also suggests the weekly enrichment of our microbial strain collection (CSUR) [12], and supports the quality control of laboratory activities in the search for deviations in laboratory processes. The overall structure of MIDaS is presented in Figure 1.

### 2.2. MIDaS Data Collection

The main role of MIDaS is to gather surveillance-related data from the hospital information system and the laboratory information system by weekly extraction–transform–load processes. The AP-HM consists of four public university hospitals: Timone (1307 beds), Conception (767 beds), North Hospital (793 beds), and South Hospital (421 beds). It has approximately 125,000 admissions and 1 million consultations per year. The clinical microbiology and virology laboratory performs approximately 8 million tests per year. Tests results, as well as patients and specimen information, are collected from the laboratory information system. Other data come from the hospital information systems, such as the hospital medico-economic data (Programme de Médicalisation des Systèmes d’Information, PMSI) used for patients’ death status in order to study death-associated infections. Data from other systems are also collected, such as spectra files generated by the Matrix Assisted Laser Desorption IonizationTime of Flight (MALDI–TOF) mass spectrometry instruments used for bacterial and fungal routine identification. MIDaS is therefore a data warehouse that groups together microbiological analysis results (sample number, requesting unit, sample date, type of analysis, antibiotic susceptibility test results, and antibiotic resistance phenotype) and patient information (anonymized patient identifier, age, gender, postal code of residence, anonymized identifier of hospital stay, date of hospitalization, length of stay, and death). Six million microbiological results are stored in this data warehouse, representing 240,000 antibiotic susceptibility tests, 2,300,000 samples, 850,000 patients, and nearly 1 million MALDI–TOF clinical spectra (with more than 3 million for spectra being produced for research purposes).

### 2.3. MIDaS Domain-Specific Monitoring Systems

Five domain-specific monitoring sub-systems are connected to the data warehouse for producing fully automated dashboards. Historically, EPIMIC (for EPIdemiological surveillance and alert based on MICrobiological data) is the first surveillance system that was implemented (in 2002) in our laboratory to allow monitoring of the weekly counts of clinical specimens sent by clinicians, diagnosis tests performed, and diagnosis results [13]. It has been later updated in 2013 for its integration into MIDaS. Since 2013, bacteria have been more comprehensively monitored by BALYSES (Bacterial real-time Laboratory-based Surveillance System), while SFY (Surveillance of Fungi and Yeasts) has focused on microscopic fungi and yeasts and MARSS (Marseille Antibiotic Resistance Surveillance System) has monitored antibiotic resistance patterns [14]. In addition, MALDI–TOF spectra have been used as an additional tool to support surveillance and are analyzed by the SpectraSurv system (for MALDI–TOF based surveillance) [15].

### 2.4. Data for the Lockdown Analysis

In this study, we were particularly interested by the bacterial agents identified in respiratory, blood, and urine samples during the lockdown period (that is, from week 12 (mid-march) to week 35 (end of august) 2020). We used the same period of years from 2017 to 2019 as control.

#### 2.4.1. Hospital Activities

**Figure 2:**
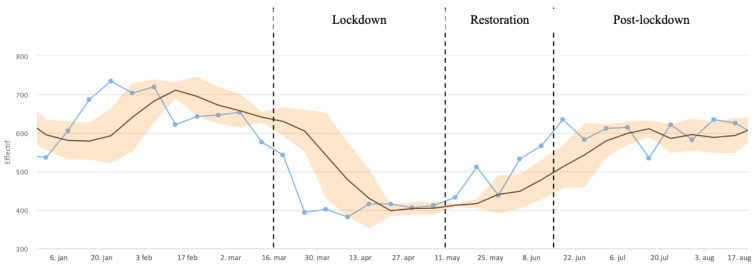
Follow-up of patients with at least one bacterial identification at IHU.

A preliminary descriptive analysis based on BALYSES automatic control boards allowed us to define three a priori periods according to the evolution of the laboratory activity during 2020: a lockdown phase (weeks 12–19), a restoration phase (weeks 20–24) and a post-lockdown phase (weeks 25–35) (Figure 2).

#### 2.4.2. Bacterial and Fungal Community

The bacterial community was studied in terms of species richness and abundance for the 3 most frequent samples: urine, respiratory, and blood samples. The specific richness represents the total number of species present in a sample and the relative abundance (or relative frequency) indicates the frequency of a species.

#### 2.4.3. Evolution of Antibiotic Resistance

We used the percentage of non-resistant (wild) isolates for each species monitored by the surveillance system without differentiating the resistance phenotypes, taking into account the nosocomial or community origin of the isolate.

### 2.5. Statistical Analysis

For further investigations of the surveillance system results, the log-linear model, and the Fisher and Chi2 tests for point comparisons, were used to evaluate the evolution of diversity and antimicrobial resistance, with a statistical significance threshold of 0.05 [16]. All statistical processes were done using software R version 4.0.3 (https://www.R-project.org, accessed on 27 May 2021.

## 3. Results

### 3.1. Hospital Activities

The follow-up of the laboratory bacterial identification activity (Figure 2) showed a drop in the moving average of the number of patients during the lockdown period from 641.5 patients on 11 March to 412.5 patients on 13 May 2020. After the end of the lockdown, the activity level gradually returned to the normal, from 412.5 patients on 13 May to 512.5 patients on 17 June and then to 611.25 patients on 15 July 2020.

### 3.2. Bacterial and Fungal Community

**Figure 3:**
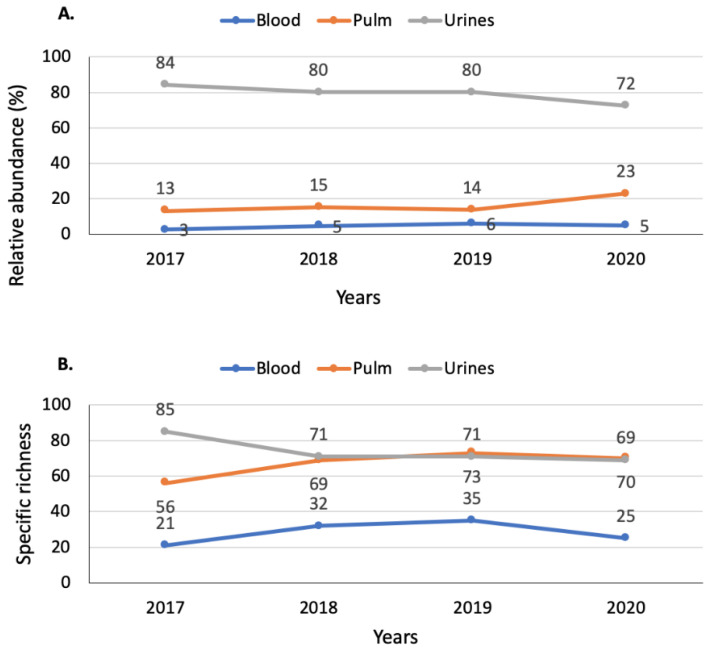
Evolution of the relative abundance (**A**) and the specific richness (**B**) from weeks 12 to 35, 2017 to 2020, at Assistance Publique—Hôpitaux de Marseille, Marseille, France.

From weeks 12 to 35 in 2017–2020, a total of 349 bacterial and fungal species were identified from 30,918 identifications including 24,946 from urine samples (186 distinct species), 4555 from respiratory samples (230 distinct species), and 1417 from blood samples (111 distinct species). The top twenty species alone represent 87.4% (27,037/30,918) of the total number of identifications. While the relative abundance in respiratory samples for the whole studied period was higher in 2020, it decreased for urine samples and was constant for blood samples (Figure 3A). However, while the species richness was constant over time in respiratory and urine samples it decreased in blood samples (Figure 3B).

**Figure 4:**
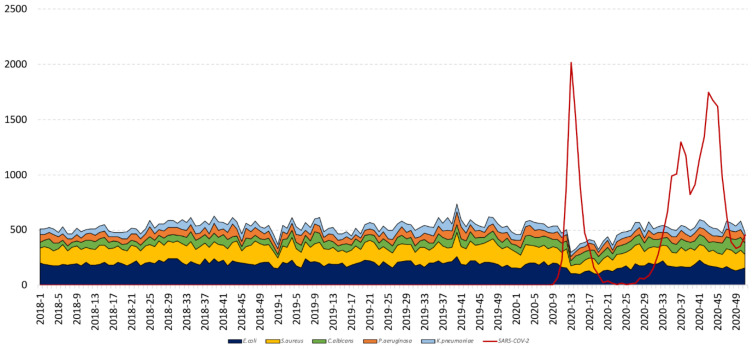
Weekly incidence of the five most identified species in our institute and SARS-CoV-2 from 2018 to 2020, Marseille, France.

When comparing diversities between 2020 and 2017–2019 for the pooled three kinds of samples (urine, respiratory, and blood samples), we found a significant variation in the relative frequency of nine species out of the top twenty (45%) during the lockdown period, and four species during the restoration and post-lockdown periods (although not for the same species) (Table 1, Figure 4). The species that significantly decreased during the lockdown were *Escherichia coli* (39.3% to 28.6%, *p*-value < 2.2 × 10^−16^), *Klebsiella oxytoca* (1.5% to 0.8%, *p*-value = 0.02), and *Haemophilus influenzae* (1.2% to 0.7%, *p*-value = 0.02). There was a significant increase of *Candida albicans*, *Staphylococcus epidermidis*, *Enterobacter cloacae, Staphylococcus haemolyticus, Enterobacter aerogenes*, and *Candida glabrata* (Table 1). *S. epidermidis* and *C. albicans* species increased during all three time periods. Conversely, *E. coli* significally decreased. *Citrobacter koseri* experienced a significant decrease only during the restoration period, and *Staphylococcus aureus* experienced significant growth during the post-lockdown (Table 1).

#### 3.2.1. Diversity in Respiratory Samples

During the lockdown and restoration periods, five species out of the top twenty recorded a significant variation in their relative frequency, and one species recorded a significant variation during the post-lockdown period (Table 2). *E. coli*, *S. pneumoniae,* and *H. influenzae* significantly decreased during the lockdown and remained stable during the next two phases. *C. albicans* is the only species that increased during the three periods. *K. pneumoniae* decreased during the restoration period, whereas species including *E. cloacae* and *S. agalactia* increased.

#### 3.2.2. Diversity in Blood Samples

A significant increase in relative frequency was observed for *E. faecalis* and *S. haemolyticus* for blood samples during the lockdown, which was maintained during the post-lockdown only for *S. haemolyticus* (Table 3). No other variation in relative frequency was observed during the restoration period.

#### 3.2.3. Diversity in Urine Samples

*E. coli* significantly decreased from 46.5% to 38.4% during the lockdown, in contrast to *C. albicans* (from 3.0% to 5.1%), *E. cloacae* (from 2.3% to 3.5%), and *C. glabrata* (from 0.6% to 1.1%) (Table 4) which significantly increased (Table 4). During the restoration period, only *C. albicans* (2.9% to 4.9%) increased and *C. koseri* (1.6% to 0.8%) decreased. No significant variation was observed for the post-lockdown period.

#### 3.2.4. Diversity in Intensive Care Units and Emergency Reception

In intensive care units, eleven of the twenty species (55%) had a significant change in relative abundance during the lockdown period. Of these eleven species, seven (63.6%) experienced a significant decrease in relative abundance (Table 5). However, the restoration and post-lockdown phases experienced, respectively, a decrease of 53% (7/13) and 58% (7/12) of their relative abundance. At the adult emergency unit, only 7/17 species (41.2%) showed a significant decrease in relative abundance compared to an increase for 10/17 species (58.8%) during lockdown. A sharp decrease in relative abundance was observed for almost all species with a significant change 7/8 (87.5%) during the post-lockdown period (Table 6).

### 3.3. Evolution of Antibiotic Resistance

Whatever the origin of the infection, the analysis of the evolution of bacterial antibiotic resistance showed a significant increase in the percentage of wild phenotypes during 2020 compared to the control period for *E. coli* (45.4% to 48.5%), *K. pneumoniae* (59.6% to 67.7%), *P. mirabilis* (56.4% to 64.1%), and *P. aeruginosa* (56.0% to 64.9%) (Table 3). The other species belonging to the 20 most represented species did not show any significant change. The wild percentage for community infection significantly increased for *E. coli* and *P. aeruginosa,* whereas it decreased for *K. pneumoniae* and *P. mirabilis*. However, for nosocomial infection, this percentage significantly decreased for only *P. aeruginosa* and increased for *K. pneumoniae*.

However, regarding the infection origin (nosocomial or community), the percentage of wild phenotypes significantly decreased when the origin was nosocomial and significantly increased when the origin was community for *E. aerogenes, E. faecium, K. oxytoca,* and *M. morganii* (Table 7). *E. faecalis* presented a decreasing percentage for nosocomial infection and *E. cloacae* an increasing percentage for community infection.

## 4. Discussion

The consequences of COVID-19 on the diversity of non-viral infectious agents and on the evolution of antibiotic resistance is notable.

The overall diversity analysis shows that the main changes were within pulmonary samplings, which were characterized by an increase of the number of different species identified in association with an increase of the species distribution evenness. Blood samples were also affected, albeit with less different species. No evident change can be observed for the urine samples.

As we worked on species’ relative frequencies for taking in account the shrinking of hospital admissions during the lockdown, the results must be interpreted in terms of species replacements. That is, the decrease of a species or group of species’ relative frequency is associated with the increase of the relative frequency of another group.

Considering the overall species’ distributions, *E. coli* was characterized by a decreasing of its frequency on the whole study period, while *C. albicans* and *S. epidermidis* showed an increase during the same period. The other species show only temporary changes, such as an increase only during the lockdown for *S. haemolyticus* or *E cloacae*, or a decrease during this same period for *K. oxytoca* or *H. influenzae*.

When considering the kind of sample we can observe that, if *E. coli* presents a homogenous global decrease, this global behavior cannot be retrieved in the sample related results. We may think that this is an example of Simpson’s paradox [17]. On the contrary, *C. albicans* and *C. glabrata* exhibited a homogenous increase on all sample related results, just as on the global one.

Beside *E. coli*, the only species decreasing in respiratory samplings, and only during the lockdown, were *H. influenzae* and *S. pneumoniae*. It is interesting to observe that these two species are well known viral co-infections, frequently observed in association with the influenza virus, and capable of coexisting in the same biofilm [18]

Few alterations of the blood-related bacterial mix were observed, concerning only *E. faecalis* and *S. haemolyticus*. They are considered to be important nosocomial pathogens, but the same behavior cannot be observed for other well-known nosocomial pathogens such as *S. aureus* or *S. epidermidis*.

More frequency changes can be observed in emergency than in intensive care units. For only one species, *P. aeruginosa*, the changes are the same, i.e., a decrease during lockdown followed by an increase. For several species (*C. albicans*, *S. epidermidis*, *S. haemolyticus*, and *K. pneumoniae*) the changes are inversed between the two kinds of units. Frequency changes only for intensive care units are specific of *H. influenzae* (decrease followed by an increase) and *S. pneumoniae* (increase followed by a decrease), while changes for *E. faecalis* (decrease followed by an increase), *S. saprophyticus*, *M. morganii* (in both cases, an increase followed by a decrease), and *S. agalactiae* (increase) were observed only in emergency units. Contou and al. [19] observed the same species behaviors in their ICU, at the exception of *H. influenzae*, which decreased in our series. When considering the global frequencies of Candida albicans and glabrata, a first hypothesis explaining their increase may be the relative importance of the admissions in ICU during the lockdown and the immediate period after.

*Candida albicans* and *glabrata* showed a significant increase of their relative frequency, whatever the type of sample and the period studied. This behavior is found in intensive care units, which were heavily impacted during the SARS-CoV-2 epidemic, and not in emergency units, possibly explaining their significant increases during the lockdown [20,21].

The wild phenotype population has also increased in comparison with the previous three years for the twenty most identified species in our institute. *E. coli* and *P. aeuruginosa* present more frequently a wild phenotype than usually in the context of community-acquired infection. However, the susceptibility of *E. coli* to most antibiotics involved in community-acquired urinary tract infections tended to decrease before the COVID-19 pandemic [22].

In France, government responses taken to limit the spread of the virus, such as lockdown measures, probably played a role in the evolution of the identification of bacteria and fungi [2,3]. Indeed, it was recommended that individuals should stay at home and contact the emergency call center (number 15) only in the event of respiratory distress in order to avoid congesting hospital resources and to prevent the spread of the disease [23]. In addition, in order to manage patients with COVID-19, many hospital departments have been transformed to accommodate these SARS-CoV-2 positive patients, which explains this increase in the number of hospitalizations. Non-emergency hospital activities were suspended. Thus, the number of patients and ordinary hospitalization outside of COVID-19 decreased considerably during the first containment, partly explaining this decrease in some pathogens and the increase in others.

## 5. Conclusions

The systematic use of a surveillance system, such as the MIDaS syndromic collection and the surveillance system at IHU-MI, made it possible to detect aberrations in the epidemic signal, to observe and analyze unexpected increases in observed cases, to implement actions to stop the spread of a pathogen, but also to understand the underlying mechanisms of its transmission. This study shows that a such system allowed us to detect and analyze the consequences of the lockdown on the bacterial and fungal population identified within our patients. Bacterial populations usually associated with seasonal viral infection where drastically reduced, while a usual raising of infections associated with *C. albicans* and *glabrata* emerged, possibly due to the increase of patients admitted in ICU.

## Figures and Tables

**Table 1:** Evolution of the relative frequency (abundance) during the lockdown period 2020 vs. 2017–2019 for the three types of sampling, Marseille.

N	Species	During Lockdown (Weeks 12–19)				During Restoration (Weeks 20–24)				During Post-Lockdown (Weeks 25–35)			
		2017–2019	2020	*p*-Value	Evol	2017–2019	2020	*p*-Value	Evol	2017–2019	2020	*p*-Value	Evol
		%	%			%	%			%	%		
1	*E. coli*	39.3	28.6	<2.2 × 10^−16^	**↘**	37.8	32.2	8.2 × 10^−5^	**↘**	37.2	35.0	0.02	**↘**
2	*K. pneumoniae*	8.8	7.7	0.12	**→**	8.7	8.9	0.78	**→**	10.4	9.3	0.07	**→**
3	*E. faecalis*	6.7	7.4	0.28	**→**	6.3	6.3	0.94	**→**	6.2	5.9	0.50	**→**
4	*P. aeruginosa*	4.4	5.3	0.06	**→**	4.7	5.0	0.61	**→**	4.8	4.8	0.84	**→**
5	*C. albicans*	4.0	7.9	8.62 × 10^−14^	**↗**	3.9	6.4	4.2 × 10^−5^	**↗**	4.2	5.2	0.02	**↗**
6	*S. aureus*	3.7	4.5	0.11	**→**	3.7	4.9	0.05	**→**	3.5	4.5	0.01	**↗**
7	*S. epidermidis*	3.5	5.0	0.001	**↗**	3.3	4.7	0.02	**↗**	2.9	3.6	0.04	**↗**
8	*P. mirabilis*	2.7	2.8	0.80	**→**	2.6	2.9	0.54	**→**	3.0	3.3	0.36	**→**
9	*E. cloacae*	2.4	3.7	0.0005	**↗**	2.6	3.2	0.24	**→**	3.3	3.9	0.10	**→**
10	*S. agalactiae*	2.3	2.1	0.46	**→**	2.	1.7	0.47	**→**	2.2	2.4	0.48	**→**
11	*K. oxytoca*	1.5	0.8	0.02	**↘**	1.4	1.7	0.44	**→**	1.3	1.1	0.37	**→**
12	*E. faecium*	1.5	1.4	0.71	**→**	1.2	1.2	0.34	**→**	1.2	1.2	0.80	**→**
13	*H. influenzae*	1.2	0.7	0.02	**↘**	1.0	0.8	0.53	**→**	0.8	0.5	0.05	**→**
14	*S. haemolyticus*	1.2	2.0	0.004	**↗**	1.3	1.7	0.34	**→**	1.1	1.3	0.19	**→**
15	*C. koseri*	1.1	1.4	0.33	**→**	1.4	0.7	0.03	**↘**	1.0	1.0	0.92	**→**
16	*E. aerogenes*	1.1	1.6	0.04	**↗**	1.0	1.0	0.87	**→**	1.3	1.6	0.12	**→**
17	*S. saprophyticus*	1.0	0.8	0.49	**→**	1.1	1.1	0.77	**→**	1.0	1.2	0.51	**→**
18	*M. morganii*	0.8	1.2	0.09	**→**	1.0	0.5	0.08	**→**	0.9	1.0	0.60	**→**
19	*S. pneumoniae*	0.7	0.5	0.18	**→**	0.6	0.4	0.40	**→**	0.5	0.5	0.85	**→**
20	*C. glabrata*	0.6	1.1	0.01	**↗**	0.7	0.8	0.81	**→**	0.6	0.7	0.65	**→**
21	*Autres*	11.6	13.4	0.03	**↗**	13.5	14.0	0.58	**→**	12.5	12.2	0.62	**→**

**↗** Significant growth; **↘** Significant decrease; **→** Non-significant change; Evol = Evolution.

**Table 2:** Evolution of relative abundance for respiratory samples from week 12 to week 35, 2020, Marseille.

N	Species	During Lockdown (Weeks 12–19)				During Restoration (Weeks 20–24)				During Post-Lockdown (Weeks 25–35)			
		2017–2019	2020	*p*-Value	Evol	2017–2019	2020	*p*-Value	Evol	2017–2019	2020	*p*-Value	Evol
		%	%			%	%			%	%		
1	*E. coli*	7.5	3.3	0.001	**↘**	5.9	5.0	0.61	**→**	5.0	5.0	0.98	**→**
2	*K. pneumoniae*	5.0	4.5	0.69	**→**	5.7	2.2	0.02	**↘**	6.5	4.6	0.11	**→**
3	*E. faecalis*	2.5	3.1	0.51	**→**	1.1	3.9	0.004	**↗**	1.6	1.4	0.82	**→**
4	*P. aeruginosa*	9.0	9.6	0.69	**→**	10.0	11.1	0.61	**→**	8.6	9.4	0.57	**→**
5	*C. albicans*	7.4	17.6	2.1 × 10^−9^	**↗**	9.0	13.3	<2.2 × 10^−16^	**↗**	8.4	13.5	0.00	**↗**
6	*S. aureus*	13.8	12.2	0.40	**→**	12.3	12.2	0.97	**→**	13.4	14.9	0.36	**→**
7	*S. epidermidis*	5.0	6.9	0.11	**→**	3.7	6.8	0.04	**↗**	3.3	5.0	0.07	**→**
8	*P. mirabilis*	0.9	0.6	0.76	**→**	0.9	1.8	0.31	**→**	0.8	1.6	0.12	**→**
9	*E. cloacae*	2.6	4.7	0.03	**↗**	3.1	3.2	0.95	**→**	5.3	5.0	0.68	**→**
10	*S. agalactiae*	1.0	0.4	0.36	**→**	0.0	1.1	0.02	**↗**	0.8	0.5	0.77	**→**
11	*K. oxytoca*	1.3	0.4	0.11	**→**	1.4	1.8	0.77	**→**	1.4	1.2	0.84	**→**
12	*E. faecium*	0.6	0.0	0.19	**→**	0.4	0.0	0.56	**→**	0.2	0.5	0.36	**→**
13	*H. influenzae*	8.7	2.9	2.5 × 10^−5^	**↘**	7.3	3.9	0.05	**→**	6.1	2.8	0.00	**→**
14	*S. haemolyticus*	2.3	3.1	0.37	**→**	2.9	2.5	0.76	**→**	1.7	1.8	0.90	**→**
15	*C. koseri*	1.0	0.8	1.00	**→**	0.7	0.4	1.00	**→**	0.5	0.5	1.00	**→**
16	*E. aerogenes*	1.3	2.7	0.07	**→**	1.1	0.7	0.73	**→**	1.6	2.1	0.38	**→**
17	*S. saprophyticus*	0.0	0.0	1.00	**→**	0.0	0.0	1.00	**→**	0.0	0.0	1.00	**→**
18	*M. morganii*	0.5	1.4	0.06	**→**	0.4	0.0	0.56	**→**	0.8	0.5	0.77	**→**
19	*S. pneumoniae*	4.6	2.0	0.01	**↘**	4.0	2.2	0.15	**→**	3.7	3.0	1.48	**→**
20	*C. glabrata*	1.0	1.4	0.41	**→**	1.3	0.7	0.74	**→**	0.7	1.1	0.40	**→**
21	*Autres*	24.3	22.5	0.42	**→**	28.7	27.2	0.64	**→**	29.8	25.4	0.05	**→**

**↗** Significant growth; **↘** Significant decrease; **→** Non-significant change; Evol = Evolution.

**Table 3:** Evolution of relative abundance for blood cultures sample from week 12 to week 35, 2020, Marseille.

N	Species	During Lockdown (Weeks 12–19)				During Restoration (Weeks 20–24)				During Post-Lockdown (Weeks 25–35)			
		2017–2019	2020	*p*-Value	Evol	2017–2019	2020	*p*-Value	Evol	2017–2019	2020	*p*-Value	Evol
		%	%			%	%			%	%		
1	*E. coli*	5.8	1.0	0.06	**→**	6.8	1.2	0.08	**→**	3.0	4.2	0.85	**→**
2	*K. pneumoniae*	3.7	1.9	0.53	**→**	2.4	2.4	1.00	**→**	5.7	3.7	0.30	**→**
3	*E. faecalis*	2.4	7.8	0.03	**↗**	1.9	0.0	0.58	**→**	2.8	4.8	0.18	**→**
4	*P. aeruginosa*	2.7	4.9	0.34	**→**	3.4	2.4	1.00	**→**	6.5	4.3	0.27	**→**
5	*C. albicans*	9.8	3.9	0.07	**→**	6.3	3.6	0.57	**→**	3.9	2.7	0.42	**→**
6	*S. aureus*	11.3	8.7	0.58	**→**	14.1	16.9	0.58	**→**	12.4	13.8	0.61	**→**
7	*S. epidermidis*	24.1	33.0	0.07	**→**	23.8	32.5	0.14	**→**	23.4	25.0	0.66	**→**
8	*P. mirabilis*	1.8	0.0	0.34	**→**	1.0	0.0	1.00	**→**	1.2	2.1	0.47	**→**
9	*E. cloacae*	2.7	1.9	1.00	**→**	3.9	4.8	0.75	**→**	4.5	5.3	0.66	**→**
10	*S. agalactiae*	0.3	0.0	1.00	**→**	0.0	0.0	1.00	**→**	0.4	0.5	1.00	**→**
11	*K. oxytoca*	0.9	1.0	1.00	**→**	1.0	0.0	1.00	**→**	0.6	0.0	0.57	**→**
12	*E. faecium*	0.6	1.0	1.00	**→**	1.0	0.0	1.00	**→**	0.6	1.1	0.62	**→**
13	*H. influenzae*	0.3	0.0	1.00	**→**	0.5	1.2	0.49	**→**	0.0	0.0	1.00	**→**
14	*S. haemolyticus*	3.4	10.7	0.003	**↗**	2.4	6.0	0.16	**→**	2.8	8.5	0.001	**↗ **
15	*C. koseri*	0.0	0.0	1.00	**→**	0.0	1.2	0.29	**→**	0.2	0.0	1.00	**→**
16	*E. aerogenes*	0.3	1.0	1.00	**→**	0.5	1.2	0.49	**→**	0.4	1.1	0.30	**→**
17	*S. saprophyticus*	0.0	0.0	1.00	**→**	0.5	1.2	0.49	**→**	0.0	0.0	1.00	**→**
18	*M. morganii*	0.0	1.0	1.00	**→**	0.0	0.0	1.00	**→**	0.0	0.0	1.00	**→**
19	*S. pneumoniae*	0.3	0.0	1.00	**→**	0.5	0.0	1.00	**→**	0.0	0.0	1.00	**→**
20	*C. glabrata*	0.3	0.0	1.00	**→**	0.5	0.0	1.00	**→**	0.6	0.0	0.57	**→**
21	*Autres*	29.3	22.3	0.17	**→**	29.6	25.3	0.46	**→**	30.3	22.9	0.05	**→**

**↗** Significant growth; **→** Non-significant change; Evol = Evolution.

**Table 4:** Evolution of relative abundance for urine samples from week 12 to week 35, 2020, Marseille.

N	Species	During Lockdown (Weeks 12–19)				During Restoration (Weeks 20–24)				During Post-Lockdown (Weeks 25–35)			
		2017–2019	2020	*p*-Value	Evol	2017–2019	2020	*p*-Value	Evol	2017–2019	2020	*p*-Value	Evol
		%	%			%	%			%	%		
1	*E. coli*	46.5	38.4	8.9 × 10^−9^	**↘**	44.7	41.6	0.07	**→**	44.1	43.4	0.53	**→**
2	*K. pneumoniae*	9.7	9.2	0.48	**→**	9.5	11.2	0.10	**→**	11.3	10.7	0.43	**→**
3	*E. faecalis*	7.7	8.7	0.17	**→**	7.4	7.4	0.96	**→**	7.1	6.9	0.70	**→**
4	*P. aeruginosa*	3.6	4.0	0.51	**→**	3.9	3.6	0.72	**→**	4.2	3.8	0.43	**→**
5	*C. albicans*	3.0	5.1	8.3 × 10^−5^	**↗**	2.9	4.9	0.002	**↗**	3.6	3.6	0.91	**→**
6	*S. aureus*	1.6	1.7	0.62	**→**	1.8	2.1	0.57	**→**	1.5	1.6	0.55	**→**
7	*S. epidermidis*	2.1	2.5	0.35	**→**	2.3	2.0	0.56	**→**	1.8	1.9	0.80	**→**
8	*P. mirabilis*	3.1	3.7	0.22	**→**	3.0	3.5	0.46	**→**	3.5	3.8	0.45	**→**
9	*E. cloacae*	2.3	3.5	0.005	**↗**	2.5	3.1	0.28	**→**	2.9	3.5	0.08	**→**
10	*S. agalactiae*	2.7	2.7	0.93	**→**	2.3	2.0	0.53	**→**	2.5	2.9	0.24	**→**
11	*K. oxytoca*	1.6	1.0	0.07	**→**	1.4	1.8	0.38	**→**	1.3	1.2	0.48	**→**
12	*E. faecium*	1.7	1.8	0.70	**→**	1.3	1.6	0.46	**→**	1.4	1.3	0.67	**→**
13	*H. influenzae*	0.0	0.0	1.00	**→**	0.0	0.0	1.00	**→**	0.0	0.0	1.00	**→**
14	*S. haemolyticus*	0.8	1.0	0.47	**→**	1.0	1.1	0.79	**→**	0.8	0.7	0.51	**→**
15	*C. koseri*	1.2	1.7	0.17	**→**	1.6	0.8	0.04	**↘**	1.1	1.1	0.95	**→**
16	*E. aerogenes*	1.1	1.4	0.35	**→**	1.0	1.0	0.99	**→**	1.3	1.6	0.27	**→**
17	*S. saprophyticus*	1.2	1.2	0.81	**→**	1.4	1.3	0.89	**→**	1.3	1.5	0.34	**→**
18	*M. morganii*	0.9	1.2	0.39	**→**	1.1	0.5	0.05	**→**	1.0	1.2	0.38	**→**
19	*S. pneumoniae*	0.1	0.0	0.61	**→**	0.1	0.0	1.00	**→**	0.1	0.0	0.59	**→**
20	*C. glabrata*	0.6	1.1	0.02	**↗**	0.6	0.8	0.43	**→**	0.6	0.6	0.80	**→**
21	*Autres*	8.5	10.2	0.03	**↗**	10.2	9.9	0.75	**→**	8.9	8.7	0.82	**→**

**↗** Significant growth; **↘** Significant decrease; **→** Non-significant change, Evol = Evolutio.

**Table 5:** Evolution of relative abundance in intensive care units from week 12 to week 35, 2020, Marseille.

N	Species	During Lockdown (Weeks 12–19)				During Restoration (Weeks 20–24)				During Post-Lockdown (Weeks 25–35)			
		2017–2019	2020	*p*-Value	Evol	2017–2019	2020	*p*-Value	Evol	2017–2019	2020	*p*-Value	Evol
		%	%			%	%			%	%		
1	*E. coli*	10.9	8.6	0.2	**→**	9.2	4.6	0,01	**↘**	15.3	13.4	0.27	**→**
2	*K. pneumoniae*	13.6	7.8	0.003	**↘**	0	0	1	**→**	8.1	5.5	0.04	**↘**
3	*E. faecalis*	0.8	2.8	0.003	**→**	4.3	0	0.0001	**↘**	6.8	7.4	0.64	**→**
4	*P. aeruginosa*	6.0	0	7.30 × 10^−7^	**↘**	10.7	17.4	0.002	**↗**	7.1	2.6	7.50 × 10^−5^	**↘**
5	*C. albicans*	4.1	10.4	3.40 × 10^−6^	**↗**	8.3	0	7.30 × 10^−8^	**↘**	8.7	19.3	2.70 × 10^−11^	**↗**
6	*S. aureus*	3.0	24.8	<2.2 × 10^−16^	**↗**	23	0.6	<2.2 × 10^−16^	**↘**	10.1	8.6	0.3	**↘**
7	*S. epidermidis*	9.5	4.3	0.001	**↘**	3.5	34.9	<2.2 × 10^−16^	**↗**	7.2	3.1	0.0003	**↘**
8	*P. mirabilis*	1.1	0.3	0.2	**→**	0.1	2.1	0.001	**↗**	2.4	1.6	0.25	**→**
9	*E. cloacae*	2.1	3.8	0.07	**→**	8.5	3.4	0.002	**↘**	5.7	7.9	0.05	**→**
10	*S. agalactiae*	0.8	1	0.75	**→**	0.2	0	1	**→**	0.8	0.3	0.38	**→**
11	*K. oxytoca*	1.4	0.3	0.09	**→**	1.7	0.3	0.08	**→**	1.2	2.8	0.01	**↗**
12	*E. faecium*	2.4	0.3	0.01	**↘**	1.4	0	0.02	**↘**	1	1.9	0.09	**→**
13	*H. influenzae*	7.7	0.3	<4.5 × 10^−8^	**↘**	3.6	6.4	0.03	**↗**	0.8	0	0.03	**↘**
14	*S. haemolyticus*	1.8	0	0.01	**↘**	0.5	1.2	0.23	**→**	0.5	3.6	5.90 × 10^−9^	**↗**
15	*C. koseri*	1.1	0	0.04	**↘**	0.4	0	0.56	**→**	0.8	1.7	0.05	**↗**
16	*E. aerogenes*	2.5	5.6	0.003	**↗**	0.7	0.9	0.71	**→**	1.7	0.3	0.02	**↘**
17	*S. saprophyticus*	0	0.3	0.26	**→**	0	0	1	**→**	0.1	0	1	**→**
18	*M. morganii*	0.2	0.3	1	**→**	0	0	1	**→**	0.8	1.9	0.03	**↗**
19	*S. pneumoniae*	2.8	5.6	0.01	**↗**	2	0	0.01	**↘**	0.5	0.2	0.47	**→**
20	*C. glabrata*	2.0	1.3	0.32	**→**	0.1	1.5	0.01	**↗**	1	0.5	0.27	**→**
21	*Autres*	26.3	22.5	0.14	**→**	21.9	73.4	<2.2 × 10^−16^	**↗**	80.5	17.2	<2.2 × 10^−16^	**↘**

**↗** Significant growth; **↘** Significant decrease; **→** Non-significant change, Evol = Evolutio.

**Table 6:** Evolution of relative abundance emergency units from week 12 to week 35, 2020, Marseille.

N	Species	During Lockdown (Weeks 12–19)				During Restoration (Weeks 20–24)				During Post-Lockdown (Weeks 25–35)			
		2017–2019	2020	*p*-Value	Evol	2017–2019	2020	*p*-Value	Evol	2017–2019	2020	*p*-Value	Evol
		%	%			%	%			%	%		
1	*E. coli*	31.0	17.7	3.50 × 10^−7^	**↘**	40.3	0.6	<2.2 × 10^−^^16^	**↘**	25.0	39.0	1.10 × 10^−14^	**↗**
2	*K. pneumoniae*	1.3	3.3	0.01	**↗**	9.5	0.0	3.60 × 10^−^^9^	**↘**	11.0	11.5	0.64	**→**
3	*E. faecalis*	8.2	0.8	5.30 × 10^−7^	**↘**	0.3	13.6	<2.2 × 10^−^^16^	**↗**	2.3	2.6	0.58	**→**
4	*P. aeruginosa*	5.9	2.2	0.004	**↘**	2.8	8.0	1.90 × 10^−^^5^	**↗**	5.2	6.0	0.36	**→**
5	*C. albicans*	7.8	0.6	3.80 × 10^−7^	**↘**	2.2	26.0	<2.2 × 10^−16^	**↗**	3.7	3.5	0.82	**→**
6	*S. aureus*	5.5	14.4	2.70 × 10^−9^	**↗**	7.8	16.3	3.10 × 10^−6^	**↗**	6.1	0.7	2.70× 10^−^^11^	**↘**
7	*S. epidermidis*	8.1	14.1	0.0004	**↗**	7.1	0.0	4.50 × 10^−^^7^	**↘**	5.7	7.5	0.06	**→**
8	*P. mirabilis*	1.9	0	0.008	**↘**	0.6	0.0	0.36	**→**	3.9	4.2	0.7	**→**
9	*E. cloacae*	0.8	0.8	1	**→**	0.3	7.7	<2.2 × 10^−16^	**↗**	4.6	1.3	9.50 × 10^−6^	**↘**
10	*S. agalactiae*	0.2	4.7	3.10 × 10^−10^	**↗**	1.1	0.6	0.54	**→**	2.3	0.1	1.40 × 10^−5^	**↘**
11	*K. oxytoca*	1.3	2.8	0.04	**↗**	0.8	0.0	0.13	**→**	1.2	0.3	0.02	**↘**
12	*E. faecium*	1.2	0	0.04	**↘**	0.5	0.0	0.35	**→**	1.0	1.4	0.31	**→**
13	*H. influenzae*	0.7	0	0.23	**→**	2.3	2.4	0.98	**→**	1.3	0.1	0.002	**↘**
14	*S. haemolyticus*	2.3	4.7	0.01	**↗**	1.9	1.2	0.36	**→**	1.6	0.4	0.01	**↘**
15	*C. koseri*	1.3	0	0.02	**↘**	0.1	0.0	1	**→**	0.9	1.0	0.73	**→**
16	*E. aerogenes*	0.2	0.8	0.11	**→**	0.3	4.4	4.60 × 10^−8^	**↗**	2.4	1.5	0.13	**→**
17	*S. saprophyticus*	0.1	3.9	2.70 × 10^−10^	**↗**	1.4	0.0	0.03	**↘**	1.7	0.0	6.80 × 10^−5^	**↘**
18	*M. morganii*	0.9	2.5	0.03	**↗**	0.2	0.0	1	**↘**	0.3	0.0	0.19	**→**
19	*S. pneumoniae*	1.1	0	0.06	**→**	1.8	0.6	0.1	**→**	0.6	1.0	0.21	**→**
20	*C. glabrata*	0.4	3.3	4.90 × 10^−6^	**↗**	0.8	0.0	0.22	**→**	0.6	0.0	0.13	**→**
21	*Autres*	19.8	76.5	<2.2 × 10^−16^	**↗**	17.9	81.4	<2.2 × 10^−16^	**↗**	81.4	82.3	0.55	**→**

**↗** Significant growth; **↘** Significant decrease; **→** Non-significant change, Evol = Evolutio.

**Table 7:** Comparison of wild percentage by origin of infection 2017–2019 vs. 2020, Marseille.

Species	Global								Origin of Infection															
	2017–2019			2020			*p*-Value	Evol	Nosocomial								Community							
	W	R	%	W	R	%			2017–2019			2020			*p*-Value	Evol	2017–2019			2020			*p*-Value	Evol
									W	R	%	W	R	%			W	R	%	W	R	%		
*A. baumannii*	74	22	77.1	9	3	75.0	1.00	**→**	20	22	47.6	9	3	75	0.11	**→**	54	0	100	0	0	-	-	**→**
*E. aerogenes*	342	49	87.5	119	16	88.2	0.84	**→**	121	31	79.6	0	16	0	1.6 × 10^−^^10^	**↘**	221	18	92.5	119	0	100	0.001	**↗ **
*E. cloacae*	786	348	69.3	265	92	74.2	0.08	**→**	515	0	100	0	0	-	-	**→**	271	348	43.8	265	92	74.2	<2.2 × 10^−^^16^	**↗ **
*E. faecalis*	1987	9	99.6	348	1	99.7	1.00	**→**	1374	1	99.9	0	1	0	0.001	**↘**	613	8	98.7	348	0	100	0.06	**→**
*E. faecium*	393	60	86.8	64	5	92.8	0.16	**→**	273	42	86.7	0	5	0	5.7 × 10^−^^5^	**↘**	120	18	87.0	64	0	100	0.001	**↗ **
*E. coli*	4791	5771	45.4	1315	1398	48.5	0.004	**↗ **	0	1892	0.0	0	1398	0	1.00	**→**	4791	3879	55.3	1315	0	100	<2.2 × 10^−^^16^	**↗ **
*K. oxytoca*	408	91	81.8	103	22	82.4	0.87	**→**	148	61	70.8	0	22	0	2.2 × 10^−^^11^	**↘**	260	30	89.7	103	0	100	0.0001	**↗ **
*K. pneumoniae*	1542	1047	59.6	484	231	67.7	7.7 × 10^−^^5^	**↗ **	1027	373	73.4	484	0	100	<2.2 × 10^−^^16^	**↗ **	515	674	43.3	0	231	0	<2.2 × 10^−^^16^	**↘**
*M. morganii*	285	52	84.6	81	14	85.3	0.87	**→**	180	38	82.6	0	14	0	1.8 × 10^−^^10^	**↘**	105	14	88.2	81	0	100	0.001	**↗ **
*P. mirabilis*	532	411	56.4	177	99	64.1	0.02	**↗ **	154	287	34.9	0	0	-	-	**→**	378	124	75.3	177	99	64.1	0.001	**↘**
*P. aeruginosa*	1343	1055	56.0	432	234	64.9	4.2 × 10^−^^5^	**↗ **	901	637	58.6	0	234	0	<2.2 × 10^−^^16^	**↘**	442	418	51.4	432	0	100	0.001	**↗ **
*S. marcescens*	304	8	97.4	119	7	94.4	0.15	**→**	191	6	97.0	0	7	0	6.5 × 10^−^^10^	**↘**	113	2	98.3	119	0	100	0.24	**→**
*S. aureus*	4458	486	90.2	31	2	93.9	0.77	**→**	1455	154	90.4	0	0	-	-	**→**	3003	332	90.0	31	2	93.9	0.77	**→**
*S. agalactiae*	770	43	94.7	123	5	96.1	0.51	**→**	253	0	100	0	0	-	-	**→**	517	43	92.3	123	5	96.1	0.17	**→**

**↗** Significant growth; **↘** Significant decrease; **→** Non-significant change, Evol = Evolution; W = Wild; R = Resistance.

## Data Availability

The data from our surveillance system are not available on the public domain, but anyone interested in using the data for scientific purpose is free to request permission from the corresponding author: Hervé Chaudet (herve.chaudet@gmail.com).
